# The role of *Pistacia lentiscus* oil in the prevention of chronic rhinosinusitis recurrence during long-term therapy: a retrospective observational case-control study

**DOI:** 10.3389/falgy.2026.1911930

**Published:** 2026-07-16

**Authors:** Alexander Bertuccioli, Davide Sisti, Chiara Maria Palazzi, Annalisa Belli, Maurizio Bignami, Marco B. L. Rocchi, Giulia Monti, Giulia Di Vincenzo, Alberto Macchi

**Affiliations:** 1 Department of Biomolecular Sciences, University of Urbino “Carlo Bo”, Urbino, Italy; 2 Microbiota International Clinical Society, Turin, Italy; 3 Upload Research Center, University of Insubria, Varese, Italy; 4 ENT Clinic, ASST Settelaghi, University of Insubria, Varese, Italy

**Keywords:** chronic rhinosinusitis, nasal preparation, phytotherapy, *Pistacia lentiscus* oil, quality of life, recurrence prevention

## Abstract

**Introduction:**

Chronic rhinosinusitis (CRS) is a prevalent inflammatory disease of the upper airways, frequently characterized by recurrence and impaired quality of life. Persistent inflammation and bacterial biofilms contribute to disease chronicity. Topical plant-derived therapies may offer a preventive strategy in long-term CRS management. This study evaluated the effectiveness of a Pistacia lentiscus–based nasal medical device in reducing the impact of CRS recurrence on patients' daily life.

**Methods:**

In this retrospective, observational, single- center case-control study, 100 adult patients with chronic rhinosinusitis (CRS) and recurrent disease were identified and included from clinical records. Patients were stratified according to prior exposure to nasal drops containing ultra-fractionated Pistacia lentiscus oil in addition to isotonic saline nasal irrigation, or to isotonic saline nasal irrigation alone, according to routine clinical practice. All patients had undergone a 12-month follow-up regimen with assessments available at baseline, 30 days, and 12 months. The primary outcome was symptom severity measured using the Sino-Nasal Outcome Test (SNOT-22). Secondary outcomes included nasal cytology parameters, biofilm presence, nasal discharge, bacterial elements, supranuclear stria, and ciliary motility.

**Results:**

Of the 100 identified subjects, 92 were included in the final analysis due to loss to follow-up. In the exposed group, SNOT-22 scores showed a 40.6% reduction after the first month, compared with a 7.8% reduction observed in the control group (*p* < 0.001). Only patients exposed to Pistacia lentiscus oil demonstrated statistically significant reductions over time in nasal discharge (*p* < 0.005), biofilm presence (*p* < 0.005), and bacterial elements (*p* < 0.05), while no significant changes were observed in the control group for these parameters. Ciliary motility remained unchanged in both groups across the study period.

**Discussion:**

Overall, within the limits of this retrospective case-control design, patients exposed to Pistacia lentiscus oil in addition to standard saline irrigation showed greater improvement in patient-reported symptoms and selected cytological markers compared with patients treated with saline irrigation alone.

## 1 Introduction

Rhinosinusitis (RS) is an inflammatory condition of the nose and paranasal sinuses, which can be classified as either acute or chronic depending on whether symptoms last longer than 12 weeks. According to the definition provided by the European Position Paper on Rhinosinusitis and Nasal Polyps (EPOS), the key symptoms of chronic rhinosinusitis (CRS) are: (a) nasal obstruction, perceived as congestion or blockage; (b) excessive nasal discharge, which may occur anteriorly with the need to blow the nose repeatedly, or posteriorly as post-nasal drip, i.e., the sensation of secretions draining retro-nasally (1). These major symptoms are often accompanied by a wide range of additional manifestations of varying intensity, which patients frequently report as the most disabling due to their impact on quality of life. Such symptoms include facial pain or pressure, frontal and/or supraorbital headache, ear fullness, reduced sense of smell (hyposmia), and impaired taste (hypogeusia). For a diagnosis of CRS, clinical symptoms must be supported by objective findings, such as at least one of the following: (i) endoscopic evidence of chronic inflammation (nasal polyps, mucopurulent discharge from the meatuses, mucosal edema); (ii) radiological findings consistent with sinus inflammation. The adoption of EPOS symptom-based diagnostic criteria has enabled standardized large-scale prevalence studies and comparisons of CRS across different populations (2, 3). Within CRS, patients with nasal polyps—although fewer in number—tend to present with more severe disease and poorer treatment outcomes. This phenotype typically appears in adulthood, with an average onset between 40 and 50 years, while CRS without polyps is more common in individuals under 40 years. The classic distinction between CRS with nasal polyps (CRSwNP) and CRS without nasal polyps (CRSsNP) is based on endoscopic findings, allowing for a clear and immediate phenotypic differentiation. Each subtype shows distinctive clinical, radiological, and therapeutic characteristics, as well as different recurrence risks. CRSwNP is more strongly associated with nasal obstruction and loss of smell and taste, while CRSsNP more frequently presents with facial pain, nasal discharge, and congestion. Our understanding of CRS pathophysiology has evolved significantly in recent decades. The older concept of an anatomical problem related to impaired sinus ventilation and drainage has given way to a more complex view that emphasizes cellular and molecular mechanisms, focusing on the immunological activity and imbalance of the sinus mucosa. Inflammatory responses can generally be divided into three distinct types, characterized by specific immune cells and mediators. Identifying these profiles helps explain the clinical heterogeneity of CRS, guides therapeutic decision-making, and allows for predictions regarding outcomes and recurrence. Several studies have attempted to classify CRS according to its immunopathological profile, identifying homogeneous subgroups based on immune activity. When matched to clinical phenotypes, it becomes clear that types 1, 2, and 3 inflammation may coexist in both CRSwNP and CRSsNP, with varying degrees of expression. However, type 2 inflammation is predominant in CRSwNP, as demonstrated by histological and immunohistochemical findings of high concentrations of eosinophils, interleukin-5 (IL-5), interleukin-4 (IL-4), and interleukin-13 (IL-13) in polyp tissue. In contrast, non–type 2 inflammation (type 1 and type 3) is more frequently observed in CRSsNP. Given the growing interest in plant-derived therapies for chronic inflammatory disorders, *Pistacia lentiscus* (PIL) has emerged as a promising candidate because of its documented anti-inflammatory, antimicrobial, antioxidant, and antibiofilm activities. Among the characteristic plants of the Mediterranean maquis of Europe, Morocco, Turkey, Iraq, and Iran, *Pistacia lentiscus* (PIL) is one of the most typical. In Italy, it is especially widespread in fragile ecosystems such as Sardinia (4), where it grows along the coast up to 700 m above sea level. It is well adapted to harsh environmental conditions, dryness, and high temperatures, factors that significantly influence its genotype and its wealth of secondary metabolites (2–19). PIL is a dioecious plant, with male and female flowers on separate trees. The leaves are leathery, shiny green, and arranged alternately in pinnate whorls. The flowers are unisexual and grouped in clusters, while the fruit is a fleshy drupe that ripens in August and changes colour from red to brown depending on the degree of maturity (1). PIL is also subject to insect-induced leaf galls, particularly from aphid species such as *Slavum wertheimae* and *Baizongia pistaciae* L., which manipulate leaves to form tumorous galls that provide shelter and nutrients for their larvae (20). These galls are rich in volatile terpenes, particularly monoterpenes such as α-pinene and limonene. Their chemical composition differs markedly from that of healthy leaves, which generally have a higher sesquiterpene content (21). From an ethnopharmacological perspective, the anti-inflammatory potential of PIL is of great importance.

## 2 Materials and methods

### 2.1 Study design

This retrospective, observational, single-center, case-control study aimed to evaluate, as its primary objective, the effectiveness of the medical device Bactorinol® nasal drops (PharmExtracta S.p.A., Pontenure, Italy) on the impact of disease recurrence on patients' daily life, evaluated with Sino-Nasal Outcome Test-22 (SNOT-22) questionnaire. Patients who received isotonic saline wash plus Bactorinol® nasal drops were retrospectively compared with patients treated with isotonic saline wash alone.

Secondary endpoints included several cytological and functional parameters related to nasal mucosal health. Ciliary motility was evaluated *in vivo* using phase-contrast light microscopy and was considered normal when a persistent ciliary beat was observed for more than 10 min. Nasal secretions were also assessed as part of the clinical evaluation. The presence of biofilm was investigated through cytological sampling performed at the mucosa of the middle third of the inferior surface of the inferior turbinate. Bacterial elements were identified by light microscopic examination of May–Grünwald–Giemsa stained cytological preparations according to their characteristic morphology and staining properties. The evaluation was limited to the presence or absence of bacterial elements, without microbiological culture or molecular identification. Consequently, differentiation at the species level was not possible within the methodological framework adopted in the present study. Biofilm was identified by the characteristic appearance of cyan-coloured areas.

Additionally, the presence of the supranuclear stria, considered a cytological feature associated with epithelial maturation and mucosal repair, was examined. The absence or reduction of this structure was considered suggestive of altered epithelial maturation or mucosal damage, which may be observed in inflammatory or infectious conditions. Cytological preparations were further analyzed for the presence of bacterial elements, acknowledging that although bacteria could be detected, their morphological features did not allow species-level identification. Variations in inflammatory cellularity, including neutrophils, eosinophils, and mast cells, were also recorded. Quantification was performed by the responsible physician using a semi-quantitative 0–4 scoring system, where 0 indicated absence and 4 indicated marked abundance. These inflammatory cells are considered markers of local inflammation and are generally present only in minimal quantities in healthy individuals.

Medical records of adult patients of both sexes with a confirmed diagnosis of chronic rhinosinusitis and a Nasal Polypoid Score ≤1 were retrospectively reviewed. Eligible patients were required to have experienced at least four recurrence episodes during the 12 months preceding treatment initiation. Patients with a SNOT-22 score <10, or with immune disorders, neoplastic diseases, or neurological and/or psychiatric conditions, were excluded from the analysis.

### 2.2 Case definition and exposure assessment

This retrospective, observational, single-center, case-control study included patients with chronic rhinosinusitis (CRS) who were identified from clinical records and stratified according to prior exposure to Bactorinol® nasal drops (PharmExtracta S.p.A., Pontenure, Italy) in addition to standard isotonic saline nasal irrigation. The exposed group (*n* = 50) consisted of patients who had received isotonic saline nasal irrigation administered via a dedicated device (Rinowash®), followed by Bactorinol® nasal drops (10 drops total, 5 drops per nostril) twice daily for 30 consecutive days. After this initial treatment phase, these patients had continued a maintenance regimen consisting of isotonic saline irrigation combined with Bactorinol® nasal drops (10 drops total, 5 drops per nostril) twice daily for 15 days per month over the subsequent 11 months. The control group (*n* = 50) included patients who had received isotonic saline nasal irrigation with an identical schedule but without exposure to Bactorinol®, and therefore represented the saline-only standard care group. Placebo administration was not performed, as this was a retrospective comparison of routinely treated patients. Clinical assessments had been performed in routine practice at baseline (T0), after 30 days (T30), and at 12 months (T365). Treatment adherence was routinely assessed during scheduled follow-up visits through patient interviews and review of medical records. Patients were specifically asked about treatment continuity, and any interruptions or major deviations from the prescribed regimen were documented by the treating physician whenever reported. At each time point, patients underwent nasal endoscopy, completed the Sino-Nasal Outcome Test-22 (SNOT-22) questionnaire, and were evaluated through nasal cytology and ciliary motility analysis.

The study protocol was conducted in accordance with the Declaration of Helsinki and was approved by the relevant ethics committee (approv. N. 95, 26 June 2025, University of Urbino Carlo Bo). The data were collected with reference to the period from April 2023 to June 2025.

### 2.3 Products characteristics

The Rinowash® probe is a specific terminal for aerosol therapy of the upper airways. Connected to a traditional compressor nebulizer, it allows a complete and accurate treatment of the upper airways in 1–3 min, for the management of allergic and non-allergic rhinitis, rhinosinusitis, and nasal polyposis. Producing particles with a diameter >10 μm, it acts exclusively at the level of the upper airways, nebulizing 5 mL of solution in one minute. Technical specifications: operating pressure 1–3.5 bar, operating flow 5–20 L/min, MMAD 18 μm. Bactorinol® nasal drops (PharmExtracta S.p.A., Pontenure, Italy) is a class IIa medical device containing ultra-fractionated and winterized *Pistacia lentiscus* oil as the active component. Bactorinol® is useful in cases of nasal congestion: it promotes fluidification of nasal secretions to relieve a stuffy nose and facilitate breathing; thanks to its emollient components, it soothes the nasal mucosa irritated due to inflammatory states from colds.

### 2.4 Statistical analysis

The Sino-Nasal Outcome Test (SNOT-22) is a validated questionnaire used to assess the severity of sino-nasal symptoms and their impact on quality of life. It consists of 22 items, each rated on a 5-point Likert scale. It allows for the evaluation of the severity of nasal symptoms, sleep disturbances, fatigue, and emotional effects, and is widely used to monitor symptom progression and treatment effectiveness, including in patients with chronic rhinosinusitis (22). In this retrospective case-control study, SNOT-22 questionnaires had been routinely administered by treating physicians during standard clinical follow-up visits as part of usual care and were subsequently retrieved from medical records for analysis. The assessment of additional clinical and cytological parameters was also performed in routine practice by the physicians, including ciliary motility evaluated *in vivo* using phase-contrast light microscopy, nasal secretion (presence/absence), biofilm detection through cytological sampling, supranuclear stria (presence/absence), and bacterial elements (presence/absence). At each time point, clinicians also documented any adverse events reported by patients during follow-up visits. Baseline significance testing for the variables age, recurrences in the previous 12 months, and baseline SNOT-22 score was performed using an independent-samples *t*-test. To evaluate the effect of Bactorinol®-exposure on the improvement of chronic rhinosinusitis symptoms, a repeated-measures ANOVA model was applied, with the total SNOT-22 score as the dependent variable and group and sex as between-subjects factors. Age was included as a covariate. Interaction terms time × group and time × age were also added. Moreover, *post-hoc* contrast analyses were performed using the simple contrast method, with T_0_ set as the reference, in order to highlight significant differences in SNOT-22 scores between baseline (T_0_) and follow-up time points (T_30_, T_365_). Within-group differences for the binary variables ciliary motility, nasal discharge, biofilm, supranuclear stria, and bacterial elements were investigated using the Cochran–Armitage test for trend (23). All tests were performed using Excel (Microsoft 365 MSO, 2512 Build version) and Rstudio (version 4.4.2).

## 3 Results

### 3.1 Participants characteristics

A total of 100 subjects with chronic rhinosinusitis (CRS) were identified and included in the initial retrospective case-control cohort. During follow-up, 8 subjects were lost to the final assessment; therefore, 92 subjects were included in the final analysis. Females accounted for 50% of the analyzed sample (*n* = 46). Baseline demographic and clinical characteristics were comparable between the exposure (Bactorinol® nasal drops) and control (saline-only) groups, with no statistically significant differences observed, as reported in Table 1. These findings support baseline homogeneity between groups in this retrospective cohort.

**Table 1:** Baseline characteristics stratified by group. The last column reports the corresponding *p*-values from the *t*-test analysis and the related Cohen's d effect size.

Variable	Exposed	Control	*p value* (Cohen's d)
Age (years)	44.2 ± 10.9	45.3 ± 12.4	0.675 (0.08)
12-month pre-study recurrences	4.4 ± 0.7	4.5 ± 0.6	0.582 (0.18)
SNOT-22 baseline score	35.5 ± 7.7	33.45 ± 7.2	0.111 (0.32)

**Figure 1:**
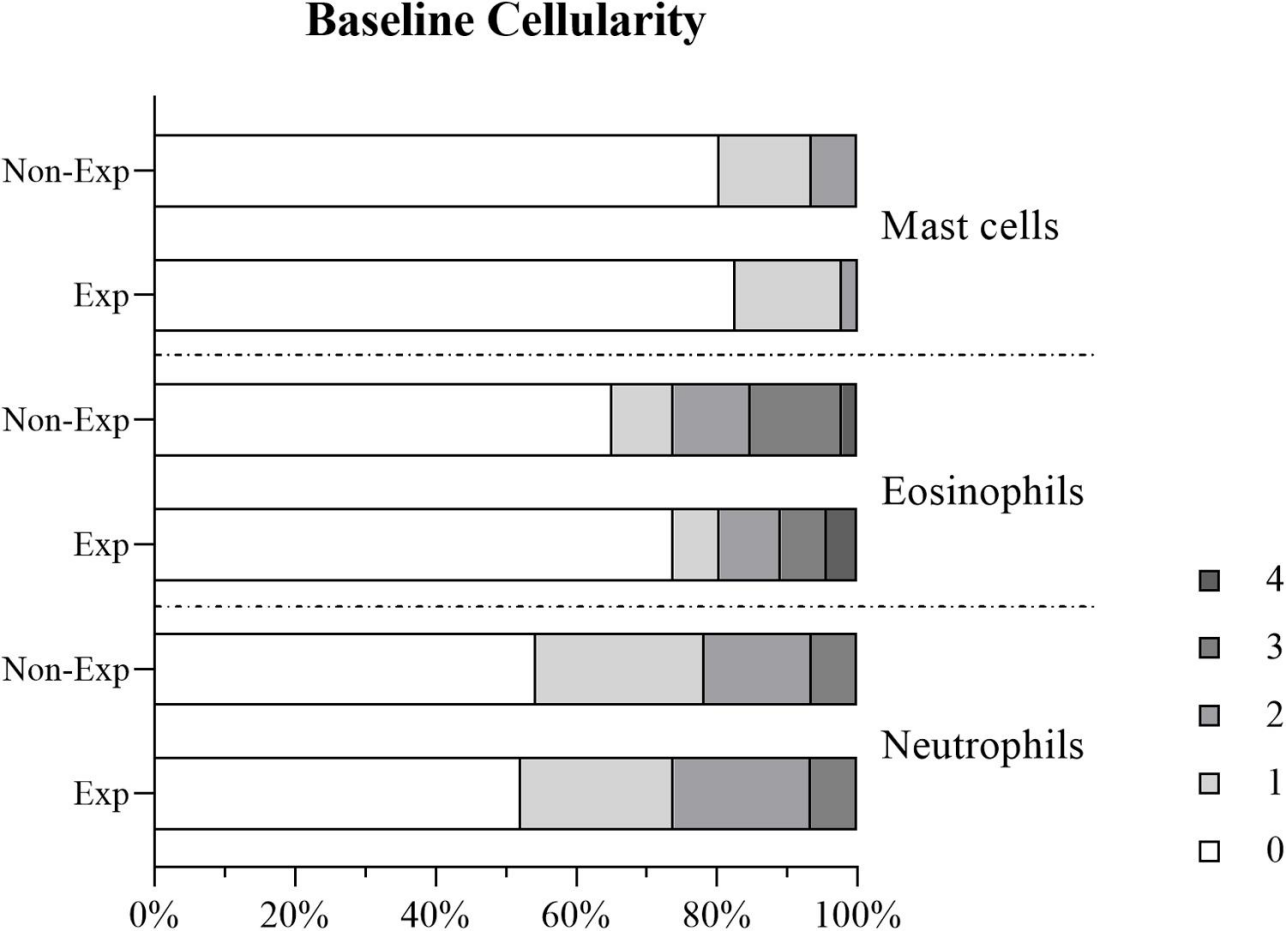
Baseline cellularity for neutrophils, eosinophils, and mast cells. The percentage reported on the *x*-axis refers to the proportion of patients.

Cytological analyses of nasal mucosal samples, retrieved from routinely collected clinical examinations, were used to assess inflammatory cellularity. Specifically, the presence and relative abundance of neutrophils, eosinophils, and mast cells were evaluated. Overall, these inflammatory cell populations showed similar distributions between the exposure and control groups, as illustrated in Figure 1.

### 3.2 *Pistacia lentiscus* oil effect

**Figure 2:**
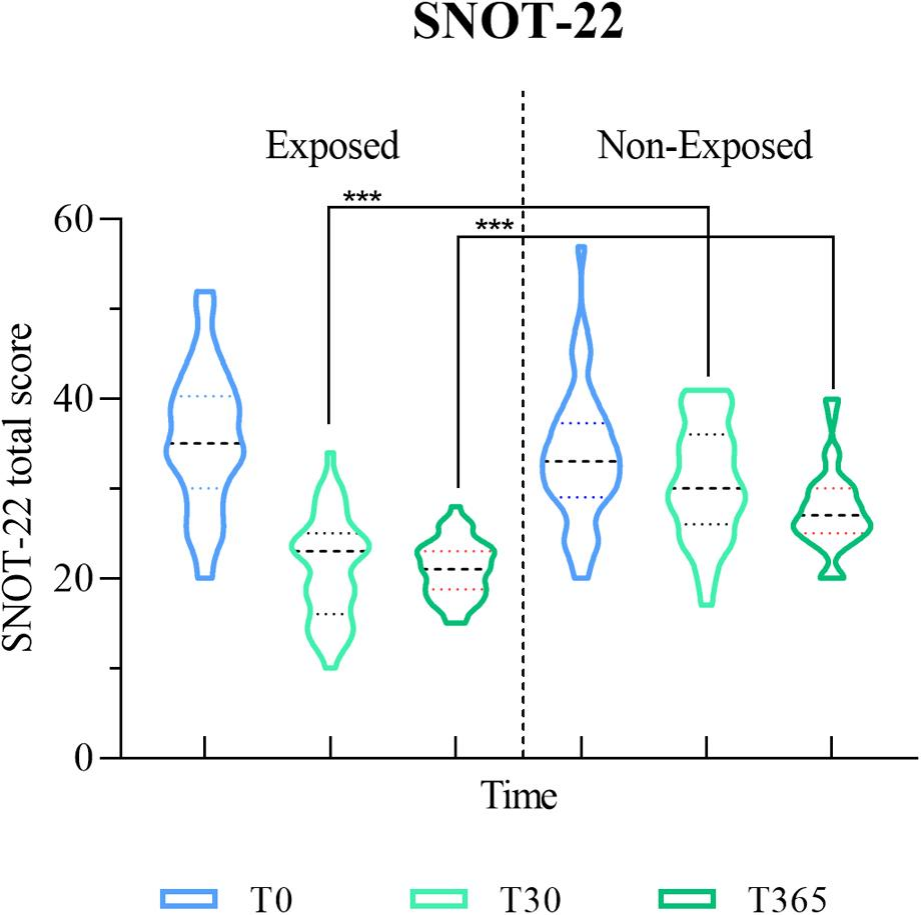
Violin plots of the SNOT-22 total score. T_0_ differs significantly from both T_30_ and T_365_ in both groups. The thick black dashed lines represent the median of the data. The thin dashed lines, instead, represent the first and third quartiles.

To evaluate the association between exposure to treatment and improvement in chronic rhinosinusitis symptoms, a repeated-measures ANOVA model was applied. The ANOVA analysis revealed highly significant effects for time (F = 9.307, *p* < 0.001) and for the time × group interaction term (F = 42.36, *p* < 0.001), indicating that although the SNOT-22 questionnaire score decreases spontaneously over time, the treated group shows a markedly greater and significantly larger reduction compared with the control group. In contrast, the time × age interaction term was not statistically significant (F = 0.420, *p* = 0.658), suggesting an age-independent effect. Contrast analyses further showed that baseline (T_0_, used as the reference) differed significantly from both T_30_ (*p* < 0.001, 95% CI: 7.31–9.79) and T_365_ (*p* < 0.001, 95% CI: 8.75–11.57), indicating that improvement is maintained up to the final time point (T_365_) with no return to baseline values, irrespective of group. As shown in the violin plots in Figure 2 and in Table 2, the SNOT-22 total score decreases over time.

**Table 2:** SNOT-22 total score at the three time points, with corresponding mean percentage changes. Values at T_0_, T_30_, and T_365_ are expressed as mean ± standard deviation.

Group	T_0_	T_30_	T_365_	Δ_T30−T0_%	Δ_T365−T0_%
Exposed group	35.5 ± 7.7	21.1 ± 5.6	21.1 ± 3.1	−40.6%	−40.6%
Control group	33.4 ± 7.25	30.8 ± 6.7	27.6 ± 4.5	−7.8%	−17.4%

**Figure 3:**
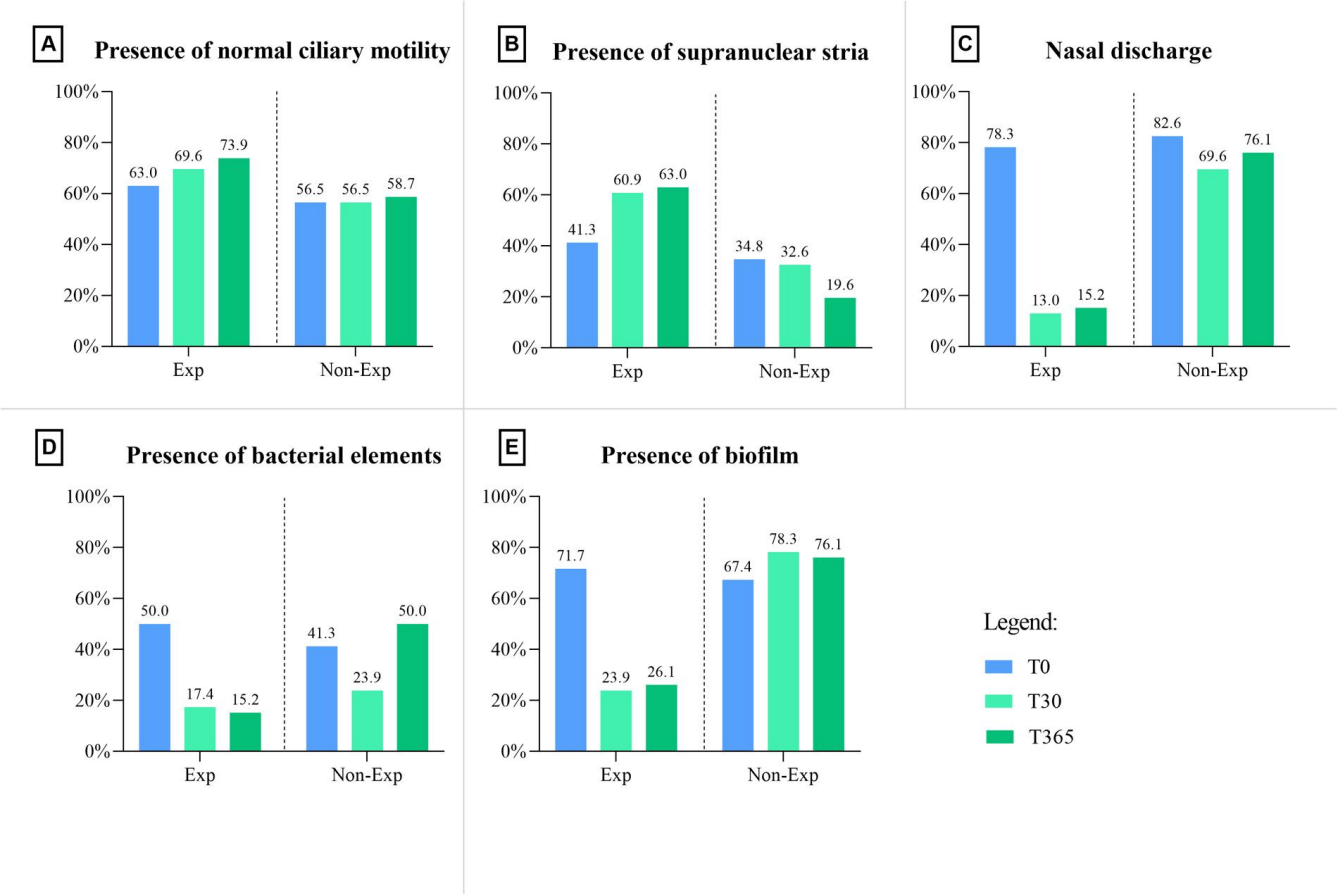
Grouped histograms showing the presence of normal ciliary motility **(A)**, presence of supranuclear stria **(B)**, nasal discharge **(C)**, presence of bacterial elements **(D)** and presence of biofilm **(E)**. The *y*-axis represents the percentage of patients with the corresponding condition.

The analysis of the previously mentioned binary variables (ciliary motility, nasal discharge, biofilm, supranuclear stria, and bacterial elements) using the Cochran–Armitage test yielded interesting results. The variables nasal discharge (z = 5.369, *p* < 0.005), biofilm presence (z = 4.459, *p* < 0.005), supranuclear stria (z = 2.096, *p* < 0.05), and bacterial presence (z = 3.734, *p* < 0.005) showed significant differences across the three time points exclusively in the exposure group, indicating a time-dependent change in outcomes (presence/absence) for these variables. In the control group, none of these differences were significant, suggesting stability of the binary responses across time points. Ciliary motility also appeared unchanged both between groups and over time (*p* = 0.26). Differences in the presence of these conditions between the two groups are shown in Figure 3. Relevant findings concern nasal discharge and biofilm presence, which show a clearly different pattern between the treated and control groups over time. In the exposure group, nasal discharge is high at baseline but dramatically decreases after 30 days, with this improvement remaining stable at 1 year. This indicates a rapid and sustained clinical benefit. In contrast, the control group shows persistently high values across all time points, with no meaningful or lasting improvement. A similar, and even more pronounced, trend is observed for biofilm presence. In the exposed group, biofilm prevalence markedly decreases at 30 days and remains low after 12 months, suggesting both short-term efficacy and long-term prevention of biofilm reformation. Conversely, in the control group, biofilm prevalence not only fails to decrease but tends to increase over time, indicating disease persistence or progression. Overall, within the limitations inherent to a retrospective observational design, these findings suggest a more favorable temporal profile of inflammatory and microbiological markers in patients exposed to Bactorinol®, compared with those receiving saline irrigation alone.

## 4 Discussion

Clinically, CRSwNP is associated with higher morbidity due to the greater severity of disease, its strong tendency to recur, and the consequent need for repeated surgical procedures and frequent exposure to medications, especially antibiotics and systemic corticosteroids. In this context, the present findings suggest that the addition of ultra-fractionated and winterized *Pistacia lentiscus* oil to standard saline irrigation may provide a clinically meaningful benefit in patients with recurrent CRS. Patients exposed to *Pistacia lentiscus* oil showed a greater and sustained reduction in SNOT-22 scores compared with controls, together with improvements in nasal discharge, bacterial elements, biofilm presence, and supranuclear stria.

These results are consistent with the anti-inflammatory, antimicrobial, antioxidant, and antibiofilm properties of *Pistacia lentiscus* described in the Introduction. Rather than indicating a single mechanism of action, the observed changes may reflect a combined improvement of the local mucosal environment, including reduced inflammatory burden, lower bacterial persistence, and improved epithelial maturation and mucosal repair. The biological plausibility of these findings is supported by previous evidence showing that *Pistacia lentiscus* contains bioactive terpenes and polyphenols with antioxidant and anti-inflammatory properties, including modulation of oxidative stress and inflammatory signaling pathways (7, 8, 11, 24–26). In addition, previous studies have reported antimicrobial activity of *Pistacia lentiscus* oils and extracts against several bacterial and fungal species, supporting their potential relevance in conditions characterized by microbial persistence (4, 9, 17). The biological activity of *Pistacia lentiscus* oil has also been attributed to the synergistic action of its terpene-rich phytocomplex, including compounds such as α-pinene, terpinene, caryophyllene, limonene, and myrcene (2).

In CRS, recurrence remains one of the main clinical challenges, as it significantly worsens patients' quality of life and is linked to persistent local inflammation, impaired mucosal function, bacterial persistence, and host-related factors (5). The reduction in biofilm presence observed in the exposed group is therefore clinically relevant, as biofilms are recognized contributors to CRS persistence and recurrence. Their resistance to host defenses and antimicrobial treatments may help maintain chronic inflammation and recurrent symptoms, while antibiotic treatment may reduce symptoms related to planktonic bacteria without fully eradicating the biofilm reservoir (16, 17). Therefore, the marked decrease in biofilm presence after exposure to *Pistacia lentiscus* oil may partly explain the sustained improvement in patient-reported outcomes. This interpretation is also consistent with previous observations suggesting that *Pistacia lentiscus* derivatives may interfere with biofilm organization and bacterial persistence (27). The reduction in biofilm presence observed in the exposed group is clinically relevant, as biofilms are recognized contributors to CRS persistence and recurrence. Their resistance to host defenses and antimicrobial treatments may help maintain chronic inflammation and recurrent symptoms. Therefore, the marked decrease in biofilm presence after exposure to *Pistacia lentiscus* oil may partly explain the sustained improvement in patient-reported outcomes.

At the cellular level, the increase in supranuclear stria observed in the exposed group may suggest improved epithelial maturation, mucosal repair, and restoration of nasal epithelial integrity. This finding, together with the reduction in nasal discharge, bacterial elements, and biofilm presence, supports a favorable change in the local mucosal environment. In contrast, ciliary motility did not significantly change over time in either group, suggesting that the clinical and cytological improvements observed in the exposed group were not primarily driven by measurable changes in this parameter.

Finally, from a clinical perspective, patients exposed to *Pistacia lentiscus* oil showed a statistically significant improvement in quality of life, as evidenced by the reduction in SNOT-22 scores compared with controls. However, given the retrospective observational design of the study, these findings should be interpreted as hypothesis-generating and require confirmation in prospective randomized studies.

## 5 Conclusion

The use of *Pistacia lentiscus* represents a means of supplementing medical therapies for the treatment of chronic rhinosinusitis. The constant use of nasal washes with special nasal douche has allowed us to highlight how the action at the level of nasal cellularity, ciliary function and secretions is evidenced by the results of the present study. Importantly, how the improvement of disease control led to the improvement of patients' quality of life.

The increase in supranuclear stria observed in the treated group suggests improved epithelial maturation and mucosal repair, which may reflect restoration of nasal mucosal integrity. Together with the reduction in bacterial elements, biofilm presence, and nasal secretions, these findings indicate an improvement in the local mucosal environment. Such biological changes are consistent with the significant reduction in SNOT-22 scores observed during follow-up, suggesting that improved mucosal health may have contributed to better symptom control and, consequently, to an improved quality of life. The cyclic use of *Pistacia lentiscus* derivative represents an attempt to increase patient therapeutic compliance by improving functional activity at the nasal level and to improve the quality of life of the patients, with the decrease of secretion and episode of recurrence. The compared data with control group confirm the validity of the therapy. The use of *Pistacia lentiscus* oil is more effective in the reduction of CRS impact on patient's daily life than the nasal washing habitually used by the patients during the follow up. The study showed a significant reduction on SNOT-22 score. In detail the study demonstrates the necessity to continue therapy in the patients with CRS to maintain a normal cellular stability. Continued use has made it possible to control the presence of biofilm and reduce the incidence of recurrence.

## 6 Study limitations

The retrospective, non-randomized design of this study limits causal inference and may have introduced selection bias and residual confounding despite the comparable baseline characteristics of the two groups. In addition, potentially relevant confounding factors, including concomitant treatments, environmental exposures, smoking habits, and other lifestyle-related variables, were not systematically controlled. The microbiological assessment was limited to the cytological detection of bacterial elements and did not allow species-level identification. Therefore, these findings should be considered preliminary. Future prospective randomized controlled trials with standardized adherence monitoring, larger sample sizes, and more comprehensive microbiological analyses are needed to confirm the long-term effectiveness of *Pistacia lentiscus* oil and further clarify its mechanisms of action.

## Data Availability

The raw data supporting the conclusions of this article will be made available by the authors, without undue reservation.
